# Biocompatible Nanomaterials in Food Science, Technology, and Nutrient Drug Delivery: Recent Developments and Applications

**DOI:** 10.3389/fnut.2021.778155

**Published:** 2022-01-20

**Authors:** Muhammad Modassar Ali Nawaz Ranjha, Bakhtawar Shafique, Abdur Rehman, Arshad Mehmood, Ahmad Ali, Syeda Mahvish Zahra, Ume Roobab, Ajay Singh, Salam A. Ibrahim, Shahida Anusha Siddiqui

**Affiliations:** ^1^Institute of Food Science and Nutrition, University of Sargodha, Sargodha, Pakistan; ^2^State Key Laboratory of Food Science and Technology, School of Food Science and Technology, Jiangnan University, Wuxi, China; ^3^Beijing Advance Innovation Center for Food Nutrition and Human Health, School of Food and Health, Beijing Technology and Business University, Beijing, China; ^4^Department of Environmental Design, Health and Nutritional Sciences, Allama Iqbal Open University, Islamabad, Pakistan; ^5^School of Food Science and Engineering, South China University of Technology, Guangzhou, China; ^6^Department of Food Technology, Mata Gujri College, Fatehgarh Sahib, India; ^7^Food Microbiology and Biotechnology Laboratory, North Carolina Agricultural and Technical State University, Greensboro, NC, United States; ^8^Technical University of Munich, Campus Straubing for Biotechnology and Sustainability, Straubing, Germany; ^9^German Institute of Food Technologies (DIL e.V.), (Deutsches Institut für Lebensmitteltechnik (English version: German Institute of Food Technologies)), Quakenbrück, Germany

**Keywords:** nanomaterials biocompatibility, food industry, food processing, food safety, food packaging, food labeling, nutrient drug delivery

## Abstract

Nanomaterials exist as potential biocompatible materials in nature and are being synthesized to provide extraordinary characteristics in various food industry sectors. Synthesis of biocompatible nanomaterials requires modification in the shape, density, and size of nanomaterials. Biocompatible nanomaterials are synthesized to reduce toxicity, decrease adverse effects in the gastrointestinal tract, and enhance immune response. Nanomaterials can target organs and tissues. Nanomaterials are found to be effectively compatible by interacting with functional foods and nutraceuticals. Applications of these nanomaterials are novel strategies in food industries such as food safety, food processing, food quality, food packaging, and food labeling. Various functions like detection of toxins and pathogens; production of biocompatible packaging; enhancement in color, flavor, and aroma; processing edible film, and sensing authenticity of food product are being accomplished with no toxicity. This review provides a systematic study on the biocompatibility of nanomaterials. It highlights the synthesis of biocompatible nanomaterials and advanced functions of these nanomaterials in the production area, processing industry, safety improvement, quality control, edible packaging films, biocompatibility, current developments, legislations and regulations for Nano-products, health and safety concerns, toxicity and public perceptions for use of nanomaterials.

## Graphical Abstract

Graphical abstract of functions of biocompatible nanomaterials in food industry. 
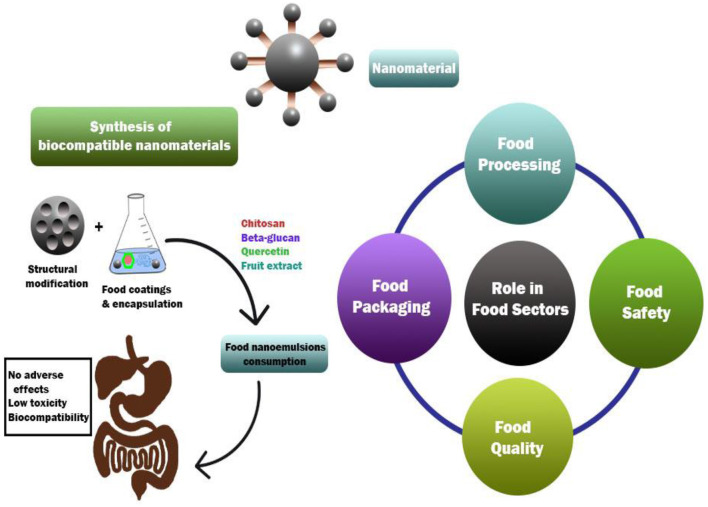

## Introduction

Innovation has always been the priority of human beings (1–3). Biocompatible nanomaterials are applied to food technology according to their properties and goals achieved. Nanomaterials have specific potentials like surface effect, quantum size effect, small size effect, and quantum tunneling effect based on physicochemical properties. These properties affect their behavior in biosystems, for they may either be tolerated or disturb biochemical and/or physiological homeostasis (4). The capability of nanomaterials to reach target tissues or organs in the organism is much more significant than their counterparts. Biocompatible nanomaterials are recognized as foreign stimuli which enhance the levels of the immune response. Naturally-occurring β-glucans has immunomodulatory property and antitumor activity. Shape, size, and surface physicochemical characteristics affect nanoparticle distribution and pharmacokinetics in the human body (5).

Nanomaterials are those materials having particles with at least one external dimension being 100 nm or with an internal structure measuring 100 nm. Nanomaterials are excellent absorbents with large surface areas despite being ultra-small, ~1–100 nm (6). Nanomaterials have diverse classifications depending on size, morphology, physical, and chemical properties. The properties of nanomaterials depend on the small grain size as they usually are pretty expressive. In the food sector, nanoparticles of zinc oxide, titanium dioxide, silica, and silver have been increasingly applied (7, 8). More novel applications of these nanoparticles are being addressed in the present era (9, 10). Nano-immune interactions should be paid special attention as immune cells recognize and engulf the particles (11).

Production of good quality food and safe evaluation by improved food sensing and good nanostructured ingredients is the aim of food processing (12–17). Silver, zinc, and calcium NPs have antimicrobial activity and biocompatibility, that's why are used as edible film (18). Generally, in food packaging and processing, nanomaterials are incorporated with polymers. The flexibility and durability of food contents get improved using these nanomaterials. The nanomaterials, along with food, enter the body by human consumption. Studying the toxicity mechanism of nanomaterials helps provide information regarding biocompatible nanomaterials (19).

Nanomaterials such as gold, nanoclays, titanium oxid, and silver are applicable in the food technology to achieve various goals like enhanced flavor, taste, appearance, shelf-life, dislikes, and likes of the consumers concerning health promising properties (20). Nanomaterials' properties could regulate physiological homeostasis and biochemical functionality. Antimicrobial properties of silver, calcium, and zinc nanoparticles are established as they are biocompatible and can be used as an edible film in food packaging (21).

Nanotechnology is being used in the production of innovative products with brand new properties. Nanomaterials of organic nature are called cellulosic nanomaterials (CNMs). CNMs are green in color, and it is possible to extract them from renewable sources, for example, wood pulp, tunicates, algae, and bacteria (22). Nanomaterials, such as metal nanoparticles, carbon nanotubes, nanostructured materials, nano-composite, and nanowires, have enhanced the biosensing and sensing design systems for applications in food analysis. Additionally, these nano biosystems are also carrying benefits in the design of novel strategies for food detection. Nanomaterials have various functions in the detection of foodborne pathogens to ensure food safety (23).

Recently, rapid progress has been made to develop the synthesized nanomaterials, although it has urged their concern of safety worldwide. In World Health Organization and Food and Agriculture Organization experts meeting in United Nations, the incredible nanomaterials' benefits in the food sectors are recognized. Nevertheless, the suggestion is given to developing the taxonomy to assist with risk management, and international and clear definitions should be revised for the nanomaterials' application (24). This review discusses the incorporation of nanomaterials into the matrices of polymers and biopolymers/natural hydrochlorides for preservation, quality, and safety purposes desirable in food science as per biocompatibility and functionalization.

## Nanomaterials in Different Sectors of Food Industry

Nanotechnology is known as multi-purpose technology that can demonstrate its application in several scientific areas at the nanoscale. Nano-technology has a wide range in providing advantages and signifies a megatrend. Scientifically, its definition is the restructuring or control of matter at the molecular and atomic levels, whose size ranges from about 1–100 nm. Advanced chemical and physical properties of nanomaterials are usually determined to provide applications that benefit society (25). Nanotechnology applications to the food and agricultural industry were first explained by the road map of the Department of Agriculture in the US in September 2003 (26).

European Nanotechnology Gateway has defined nano foods as the food in which nanotechnology techniques with nanoparticles are majorly used in harvesting, processing, and preserving food (27). The novel applications, materials, and products are expected to provide numerous improvements and advancements to the food and linked sectors, enhancing agriculture and distribution, storage, nano additives, food processing, safety, quality, and sensors to detect toxins and contaminants and innovative products' development (28).

Nano food packaging enhances the physicochemical qualities of food by reducing microbial load by acting on cell membranes and generation of reactive oxygen species and also provide physical, chemical, and mechanical resistance. Contaminants in food matrices can be isolated and pre-concentrated efficiently with the help of these nanoparticles. Furthermore, novel applications of nanomaterials include improvement in colors and flavors; detection and control of microbes, allergens, and contaminants; properties and performance of food packaging (29).

Nanomaterials are recognized for applying their technologies in food, food supplements, additives, and materials in contact with food. Internet databases' inventory reported that about 140 food products and food relevant sectors are acknowledged to claim the availability of nanomaterials in food. Application of nanomaterials has excellent diversity, containing bioactive ingredients that range from metal oxides, inorganic metal to organic nanomaterials (29). Nano-encapsulated resveratrol exhibited better anti-oxidant, anti-diabetic and anti-obesity potential than free resveratrol in the *in vitro*-digestion study, which emphasized further studies to be conducted for checking bioavailability and safety (30). The demand for the provision of healthy food is increasing day by day. Much attention should be paid to modernizing technologies of food processing to produce functional and novel food products containing enhanced benefits for health. Nano-technology has been applied to the industry of food in this context. It is widely studied to increase the biological efficacy and physicochemical stability of naturally occurring or fortified bioactive compounds during food processing (31).

## Biocompatibility of Nanomaterials

The biocompatibility of nanomaterials is the potential term that is attributed to significant safety concerns. Recently, scientists from the biological, medical, environmental, and chemical fields have drawn considerable attention to controlling and investigating the adverse impacts of nanomaterials. Several novel techniques (e.g., biomaterials gained from nature, biosynthesis, and chemical chain modification) are used to control nanotoxicity. These are also adopted to synthesize biocompatible nanomaterials with numerous sizes, functions, and shapes (e.g., quantum dots nanocrystals, nanotubes, nanowires, and nanoporous) (32, 33).

The negative impacts of nanomaterials are reduced by surface modifications, which require regulation in translocation, stability, density, and surface charge. A surface modification alters nanomaterials to determine the association between nanomaterials and biological interface. This procedure is significant to improve applications and to alleviate adverse effects. Nanomaterials are fabricated and functionalized with different moieties to decrease toxicity to human health. These moieties provide new physicochemical properties to nanomaterials with improved biocompatibility (34).

Shape, structure, and size (chemical and physical) are significant nanomaterials' elements associated with biological impacts and site of distribution and deposition within the body and clearance. Reactivity and surface charge are the parameters adapted to deliver a preferred functionality to a specified nanomaterial and influence distribution, clearance and deposition, and other inflammatory responses to the nanomaterial. Characterization of nanomaterials' physicochemical parameters is significant to the extreme possible extent to regulate the accurate dose-response materials' characteristics (35).

Nanomaterials exhibit possibilities based on physicochemical properties that regulate their performance in the biosystems to determine the cause of biochemical disturbance or homeostatic. Nanomaterials are well-established in the role to incorporate essential oils, for instance oregano oil or cinnamon for better use of edible film in the food packaging. Mostly, nanomaterials are made up of polymers and are used in food processing and food packaging. The role of better-nanostructured constituents in food sensing is the primary purpose of food processing to establish safe assessment and better food quality. These nanomaterials enhance the durability and flexibility of the contents of food. The nanomaterials can be consumed with beverages, drinks for consumption, and other food products (36).

Maintenance and improvement in health have been exhibited potentially by phytochemicals. These phytochemicals are also associated with the prevention and treatment of some diseases. Low stability, solubility, specificity, and target bioavailability somehow contain side effects when consumed at increased levels, limiting their use. Certainly, stability and solubility of phytochemicals are increased by nanoparticles with enhancement in absorption and protection from premature degradation and increase circulation time in the body. Furthermore, several nanoparticles reveal increased differential efficiency uptake in the tissue or target cells compared to normal tissue or cells by inhibiting nanoparticles from interacting prematurely with the biological environment and increasing permeation. Improvement in cellular uptake and retention impact in disease tissues and reduction in toxicity is exhibited by nanomaterials. Major biocompatible nanoparticles comprise lipid nanocarriers, poly (lactic-co-glycolic acid) nanoparticles, nanoliposomes, micelles, and nanoemulsions (37).

Starch-based nanoparticles (SNPs) have contributed much interest to protect, encapsulate, and provide bioactive components orally due to their vast ability, high biocompatibility, functionality, and environmental responsiveness. A wide variety of particle sizes can be used to synthesize SNPs with a broad range from few nanometers to hundred nanometers which approximately range from about 8–448 nm. Comparison can be made with the dimensions of nucleic acids (5–100 nm long, 2 nm wide), viruses (10–500 nm), cell organelles (5–100 mm), and proteins (1–10 nm). The ability to adjust SNPs' properties and dimensions permits them to be utilized to build biological entities complexes, thus changing their performance in function. SNPs provide improvement in the hydrophobic substances solubility and bioactive nutritional attributes. For example, bioactive bioavailability is increased by SNPs. These nanomaterials are also designed to deliver to the same gastrointestinal tract regions (38).

Cellulose nanomaterials are extracted from renewable sources and impart exceptional biocompatibility and mechanical strength. Chemical and physical properties are associated with several nano-composite materials, which is an exciting prospect for application in the food, nutraceutical, and biomedical industries. Bio-nanomaterials linked with nanotechnology have incredible potential to utilize and enhance bioactive and nutrient absorption, pharmaceutical, nutraceutical field, and drug delivery systems through numerous applications (39). Synthesis of biocompatible nanomaterials and their interaction with the human body have been reported in Table 1.

**Table 1:** Biocompatibility of nanomaterials by consuming different nanofoods products.

**Nanomaterial**	**Nanomaterial structure**	**Average size (nm)**	**Synthesis of food nanomaterial**	**Oral administration**	**Interaction with human organ/cells**	**Biocompatibility assessment**	**References**
Zinc-layered hydroxychloride	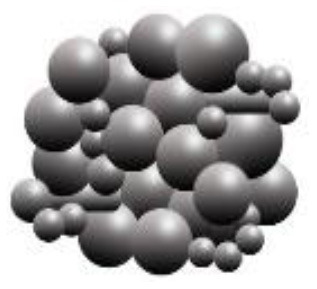	30 nm	Zinc-layered hydroxychloride coupled with yeast β-glucan	Fish spleen leukocytes	Improvement in cell viability against the bacterium *V. parahaemolyticus*, stimulate antioxidant activity	Cellular immune response was evaluated	(40)
Silver nano-particles	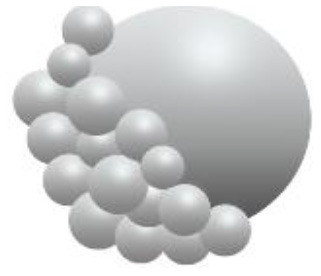	2 nm	Biosynthesis of silver nanoparticles utilizing crustacean β-glucan binding protein	Blue swimmer crab *Portunus pelagicus*	Exhibit antibiofilm property against pathogens to avoid chronic infections	Limit toxicity impact, synthesized from protein to improve biocompatibility	(41)
Gold nano-particles	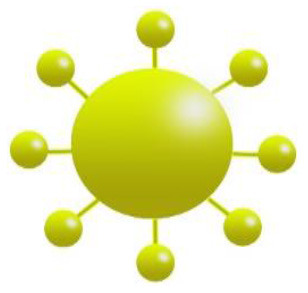	1 nm	β-glucan-based coating on gold nanoparticles	Edible mushroom *Pleurotus florida*	Enhance the growth and activity of gut microbiota, boost innate immunity	Biodistribution of nanohybrids in the gastrointestinal tract	(42)
Carbon dots	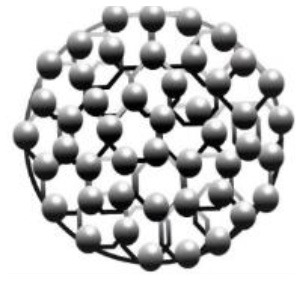	2.75 nm	Extracted from grilled pike eel	Fish *Muraenesox cinereus*	Possess physicochemical properties and health benefit	Excellent biocompatibility, low toxicity	(43)
Lipid nano-particles	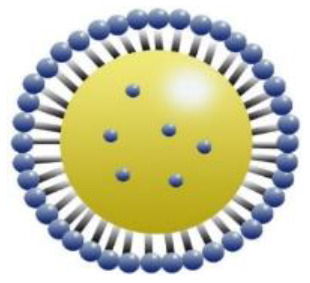	50 nm	Chitosan coating on curcumin loaded solid nanoparticles	Curcumin *Curcuma longa*	Enhance the efficacy, stability, and solubility of absorbed curcumin within the cells	Non-toxicity, biocompatibility	(44)
Chitosan/alginate nano-particles	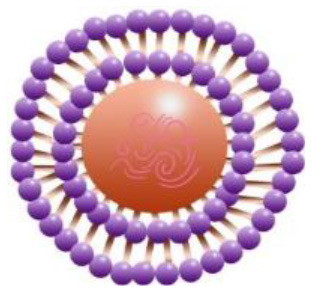	20 nm	Quercetin encapsulated in nano-particles	Natural antioxidant quercetin	Improve activity of encapsulated antioxidant	Better protection against oxidative stress, lack of toxicity	(45)
Silica nano-particles	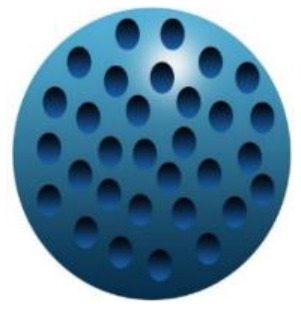	10 nm	Synthesis of biogenic silica using rice husk	Rice husk	Cellular morphological changes in human mesenchymal stem cells	Excellent biocompatibility by variable composition, structure and density	(46)
Palladium nano-particles	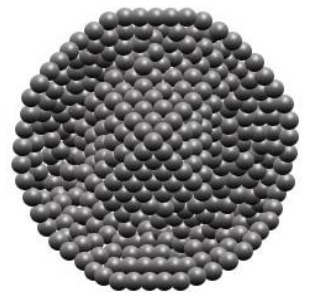	5–15 nm	Synthesized using *Couroupita guianensis Aubl* fruit extract	Aqueous fruit extract of *C. guianensis Aubl*	Destroy bacterial pathogens, exhibit anticancer properties	Safe to use in food, does not interact with red blood cells, use as multifunctional hybrid	(47)
Protein based silver nanoparticles	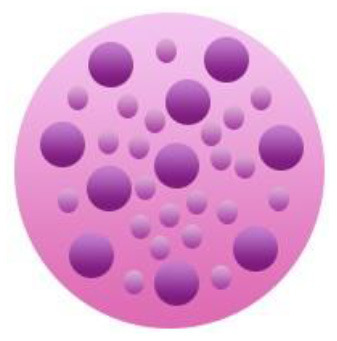	135 nm	Synthesized by full cream milk whey protein by combining with silver nanoparticles	Used in food coatings	Inhibit gram negative bacteria such as *Escherichia coli* and *Salmonella typhi* as well as gram positive bacteria *Staphylococcus aureus* and *Bacillus subtilis*	Low toxicity, effective to use as coating material, high biocompatibility, and effective to use in food products	(48)
Polysaccharide based metallic nano-particles	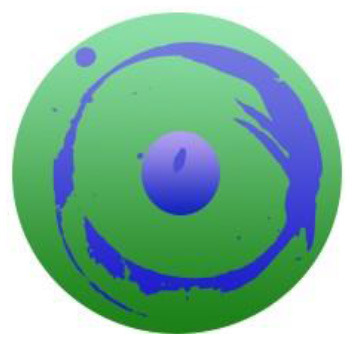	10–1,000 nm	Synthesized by the combination of gum arabic (GA) and chitosan (CS)	Used as packaging material for curcumin encapsulation	Prevent oxidation of curcumin and active for the release of nano based polysaccharide in gastrointestinal tract	Possess excellent biocompatibility characteristics	(49)

Carbon and its allotropic forms are found to be biocompatible with the human body. Carbon nanoparticles possess exclusive biological activity based on the availability of free bonds on the surface of the carbon. In the present knowledge, graphene oxide and nanodiamond nanoparticles possess no substantial toxic impacts on animals. Carbon nanoparticle biocompatibility is associated with the nanoparticle biodistribution in internal organs. Carbon nanoparticle biocompatibility is an imperative element in food packaging selection (50).

Bioactive lipids like omega-3 fatty acids, fat-soluble vitamins, conjugated linoleic acids, phytosterols, and carotenoids are degraded chemically during storage of food or within the human gut due to the exposure to several stressors, such as oxygen, high temperatures, metabolic/digestive enzymes, moisture, light, and pH which decreases their bioavailability (51–53). Physiochemical stability, bioactive lipids bioavailability, and matrix compatibility are improved by nanotechnology. Certainty, food-grade ingredients are utilized to synthesize edible nanoparticles to participate in food applications effectively. Several kinds of delivery systems on the nanoscale (such as SLNs, Pickering emulsions, nanoemulsions, biopolymer nanogels, and nanoliposomes) are found to encapsulate various kinds of bioactive lipids effectively (e.g., carotenoids, oil-soluble vitamins, essential fatty acids, and phytosterols) (54).

**Figure 1:**
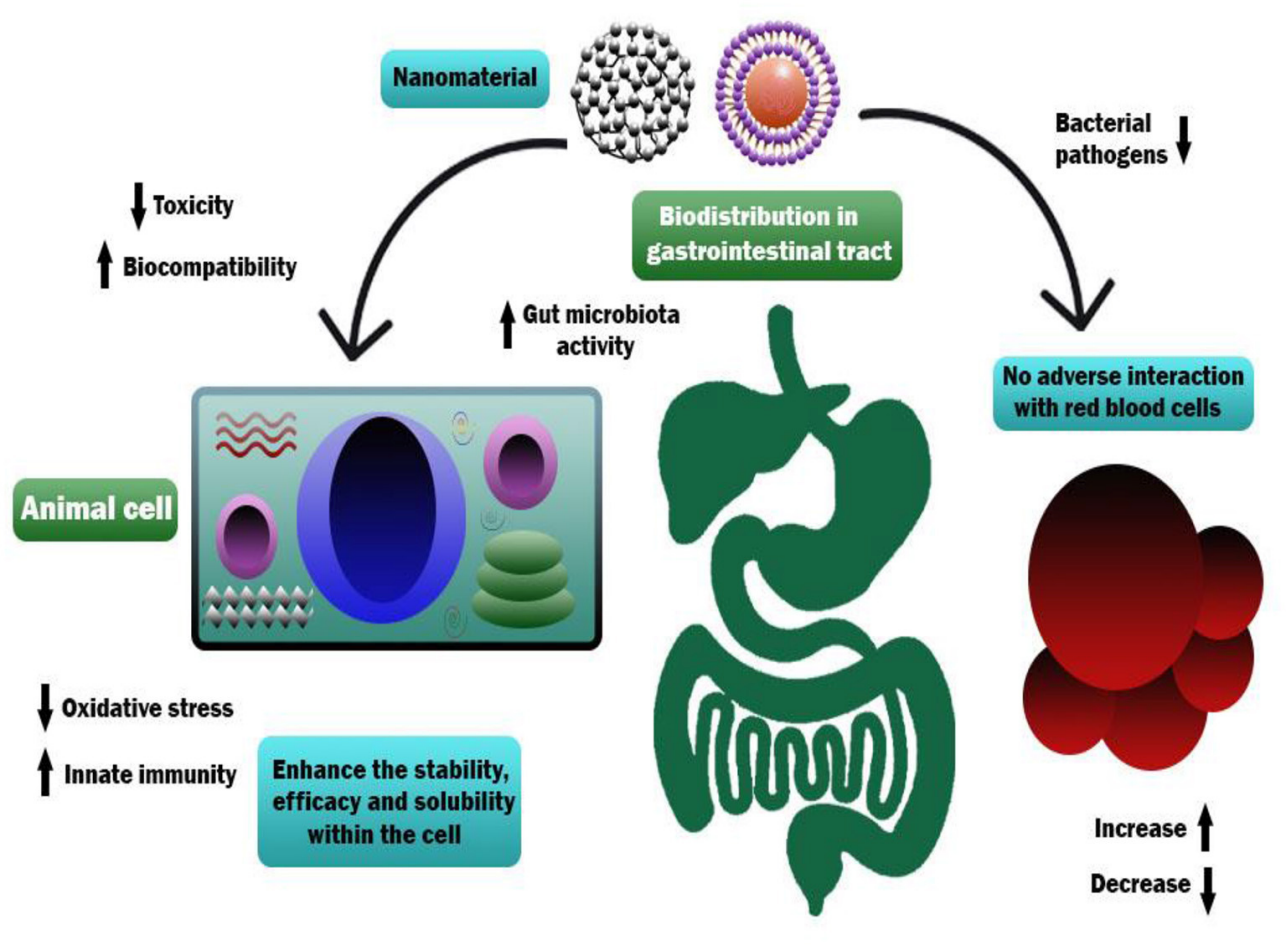
Action mechanism of various biocompatible nanomaterials in human body.

An adequate understanding of links between biological systems and nanoparticles is required to improve safety standards and design new nanomaterials generations. The intracellular fate of engineered nanoparticles, molecular, cellular uptake mechanisms, and their potential distribution in the living organism must be examined. A strategy such as binding protein and opsonization has significant prominence because they strongly influence bio-distribution, cellular internalization, and nanoparticle immunogenicity *in vivo* and *in vitro*. Therefore, it is significant to explain the molecules' mechanisms associated with cellular uptake binding and NMs processing. Accumulation of NMs in the human body could be prevented. Therefore, they should be intended to be biocompatible to control their increased toxic effects while exposed by humans (55). The action mechanism of biocompatible nanomaterials in the human body has been represented in Figure 1.

## Problems of Non-Biocompatible Nanomaterials

Sanoj Rejinold et al. have investigated drug delivery for cancer through curcumin as bioactive ingredient and temperature sensitive biodegradable chitosan-g-poly (N-vinyl caprolactam) NPs (TRC-NPs) as vehicle. Heme assay was done by taking fresh blood to investigate the compatibility of blood for bare and curcumin-loaded TRC-NPs. Results revealed heme breakdown of <5 percent, and indicted a little damage to erythrocytes, according to ISO/TR 7406 (56).

Before approving nanomedicines for clinical use, experimental safety evidence, particularly negative impacts, is required (57). Higher generation dendrimers are the most hazardous. Cytotoxicity also relies on the surface charge, with cationic dendrimers being more hazardous than anionic dendrimers, which leads to apoptosis as a result of dysfunctioned mitochondria (58, 59). Carbon nanotubes are a revolutionary kind of nano—materials with several potential uses simultaneosuly poses adverse impacts on respiratory system. Agglomerated NPs can change physicochemical characteristics, affecting biological consequences (60).

## Biocompatible Nanomaterials in Food Processing

Nanotechnology is applied in all sectors of food, including processing as well as production of functional foods, development of foods that are capable of modifying their color, flavor, or nutritional properties according to dietary needs of a person, along with the production of more assertive flavorings, colorings, and nutritional food additives (36). Moreover, this technology also lowers the costs of food additive ingredients and increases food products' shelf life (61). The food market needs technologies capable of assisting the market leadership in the industry of food processing to produce fresh, authentic, convenient, and flavorful products (62).

To obtain more information, the European Food Safety Authority (EFSA) commissioned RIKILT, and the Joint Research Center (JRC) organize a computational library of current and expected applications of NMs in the agriculture sector regarding production of feed and food as well as their safe and effective procedures to produce new food products. The primary purpose is to admire the safety concerns of nanomaterials (63–65). Currently, applications in the development of food additives from nanomaterials are prevailing. It is evident from EU and non-EU legislation that presently, the definition and specific provisions of NMs are incorporated in a few EU legal acts. In contrast, applying a broader approach provides guiding tools for the food industry in many non-EU countries (66).

Transformation in many research areas ranging from electronic and energy applications to environmental and biological disciplines is contributed by rapid development in nanomaterials synthesis, surface modification, and design. Nanomaterials have unique mechanical, chemical, biological, and physical properties. Food science is a multidisciplinary field incorporating chemistry, physics, biology, and engineering and has significant importance for life. It is an applied science dealing with food, particularly associated with the production area of food processing (67). Nanotechnology has evolved the concept of nano-phytomedicine; despite lag of regulatory standards, plant based medicines are being developed as these are naturally extracted and have no side effects so applying nanotechnology helps these to reach deep in cells, examples include drug delivery in cancer especially as nutraceuticals or functional foods (68). Neuroprotective phyto-active substances such as resveratrol, catechin, curcumin, quercetin, and ginsenosides may be bioavailable, stable, and dissolvable in the brain, but *in-vivo* investigations show their concentrations are too less to penetrate the blood–brain barrier. To circumvent these issues, nanophytomedicine with a regulated (1–100) nm size is employed (69).

Nano-technology provides enormous opportunities to enhance life quality through applied procedures in food systems and agriculture. Various advance agro- has been established, such as nano-fertilizers, biocontrol agent's nanoformulations, nano-sensors, and nano-pesticides products based on nanotechnology, which is recently an extreme investigation subject. Numerous nanomaterials have been acclaimed in agriculture for use to assist in the reduction of agrochemicals consumption by the potential use of intelligent systems of delivery, which decrease the nutrient losses and enhance the yield through optimized nutrients and water management (70).

The food processing sector has been established by the potential application of biocompatible nanomaterials in food. Nanofoods i.e., coatings, biofilm, and emulsions after production are consumed by human beings (71). Encapsulation of food additives is tremendously increasing with an interest in utilizing nanomaterials (72). The nutritional value of food is also enhanced by the possible applications of nanomaterials in fertilizers which bring a vital strategy to improve health by consuming those food products (73).

The spray drying method has been increasingly applied in food products, such as in formula milk products used by babies, soluble cocoa powder in milk and powdered sweets for children and food-based supplements rich in vitamins, proteins, and minerals for adults. These products are formulated first in solution and then transformed into powders to assist in preservation for extended shelf life. Recently, innovative technologies like nanospray drying have been developed to produce food formulations with maximum cost effective yet highly bio-active substances (74). Encapsulation and drying of several food constituents such as minerals, carotenoids, essential oils, vitamins, fatty acids, and phenolic compounds are performed by potential nano spray dryers. Nano spray drying (NSD) involves the reduction of size for the uniform distribution. Nanoparticles are rearranged and collected by applying nano-spray drying. Power dischargers and modified atomizers have been applied to increase the efficiency. NSD increases the yield of process to establish maximum attainability. Nono-encapsulated bioactive components revealed better efficacy and retention as compared to freely available ones in intestinal juice incase of catechins when studied for *in vitro*-digestion (75). Furr and Clark propose that carotenoids are taken up by bile's salt complexes, which then transfer them to chylomicrons. Direct nanoparticle absorption can theoretically increase the efficacy of dissolved but partially assimilated minerals and nutraceuticals (76). Ratnam et al. advocate using nanoscale drug delivery systems for more polar molecules like isoflavones, and micro- and maybe even nanoscale particles as the most efficient approaches (77).

Nano-technology has begun to influence many scientific fields and industries, including food technology, meaningfully. Several nanosystems such as nanoliposomes, dendrimers, liposomes, emulsions, quantum dots, and nanotubes play a potential role in food processing. The development of nanoemulsions, nano-biofilms, coatings, and packaging films is due to the efficient nanotechnology and nanofilms (78). Nanofilms protect foods from dryness. Shelf life of food products enhanced by using containers lined by nano-film coated with silica nanoparticles. Nanofilms are really good barrier to moisture, oxygen and microbes. Silicate nano-particle coating makes it water repellant due to decline in surface energy and inlined coarseness of surface, this could further be enhanced by coating of hydrophobic layer (79). Layer by layer deposition of oppositely charged layer is the best technique to formulate a good nanofilm (80). Polysaccharides, gums, polypeptides, polymers, lipids, and their aggregates having antimicrobial properties are all examples of renewable/reuseable biopolymers derived from animals and plants. Triclosan a bioactive compound incorporated in polymer basically rubber is being sold as food container with brand name of Microban, Wasabi extract is being used as coated polyethylene terephthalate film after encapsulating in cyclodextrin which can be purchased as Wasapower (81).

Essential oil-coated films which have been explored extensively include garlic oil, rosemary, cinnamaldehyde and allyl isothiocyanate (AIT) (82–85). Researchers have developed efficient nano-preservatives (NPRs) having diverse applications. However, the literature available on food preservation based on nanotechnology does not include molecular perspectives involved in food preservation. To design edible coatings, nanotechnology, and interface domain which is concerned with the physics of intermolecular and interfacial forces, contribute a lot, and there is a significant knowledge gap in this domain. To develop efficient NPRs, identification of contributing factors at nano and molecular level is needed urgently. Moreover, in terms of public interest it is important to understand the impacts of NPRs on health (86). Edible nano-coatings are now being utilized not just for shelf life improvement.

## Biocompatible Nanomaterials in Food Safety and Quality

To detect tiny amounts of contaminants, viruses, or bacteria, the development of analytical methods is another important use of nanotechnology. This will enhance the safety to the food processing system. Regulatory systems are needed urgently to manage any risks involved with nanofoods and the use of nanotechnology. Due to the growing demand for enhanced shelf life and protection from various foodborne diseases, it is expected that many areas of food science can be transformed using nanotechnology (87).

The shelf life of *Fragaria ananassa* (strawberry) and *Citrus limon* (lemon) is enhanced when Silver nano-particles in poly(vinyl alcohol) matrix (AgPVA) nanofiber is surface-coated over these fruits, AgNPs were developed by extracts from black currents peels (88). *Thymus vulgaris* (Thyme) leaf extract has been used to develop phytochemicals stabilized zinc oxide (ZnO) nanoparticles that have also pronounced antimicrobial properties against gram-negative bacteria. In recent years, a promising trend regarding the screening use of NMs as adsorbents has been observed in food safety (88, 89).

Prolonged shelf life and freshness, along with the quality of the food products, are the targets. Knowledge is increasing significantly as much work is done by biologists, chemists, technologists, physicists, and ecologists, particularly in nanotechnology; however, little is known about many aspects like the interactions with plant biomolecules. Organic and inorganic materials are used to synthesize nanomaterials, and their size is between 0.2 and 100 nm. Biocompatible nanomaterials aid in developing more innovative gadgets to meet the upcoming challenges in food technology (90).

Evaluation of various nanomaterials (NMs) in food sample pretreatment like metal-organic frameworks (MOFs) has been done. Covalent-organic frameworks (COFs), ordered mesoporous silicas (OMSs), polydopamine-derived materials (PDA), materials based on carbon, molecularly imprinted polymers, as well as other novel nanomaterials are being used for this purpose. When conventional adsorbents are compared, excellent performance is shown by the functional nanomaterials in terms of removal and pre-concentration of contaminants from food, resulting in a significant improvement in accuracy, detection, precision, selectivity, and sensitivity. Up till now, as far as biological and environmental samples are concerned, summarization of the applications of nanomaterials as solid-phase extraction adsorbents has been well-presented (21).

Nanostructures can rapidly and directly detect disease-related biomarkers, like nanotubes, nanowires, nanoparticles, microarrays, cantilevers, and nanoarrays, as part of an accurate process of sample and significantly increased sensitivity. There is the requirement of accurate techniques regarding identifying and detecting pathogenic bacteria, thus assuring food safety. A unique combination of magnetic nanoparticles and diverse carbohydrate bioactivities is needed to develop a safety system based on magnetic glyco-nanoparticle (MGNP). MGNPs are used for the first time to detect, quantify, and differentiate *E. coli* cells and can provide a new arena for applications in decontamination and food safety (91, 92).

To guarantee that food reaches the consumers in the best possible conditions in terms of freshness and microbiology, food safety and quality are considered important features. These aspects are secured and enhanced by nanotechnology. Fortification of food with bio-actives and controlled and targeted release in the gut is provided by novel technologies like nanofabrication and nanoencapsulation. Food quality can also be enhanced by the direct addition of nanomaterials into the food matrix or materials which come in contact with food (18, 93).

Incorporation of nanomaterials in packaging can be done in nano-composites by solvent casting, melt compounding, and lamination or electrohydrodynamic processing (EHDP). Active, passive, and even bioactive properties are promoted by it. These characteristics include anti-oxidation, antibacterial, barrier, and oxygen scavenging roles, and the controlled release of functional ingredients. The envisioned or non-intended movement of the nano-sized materials or the active ingredients they may transfer can be achieved. Lastly, unique opportunities in circular bio-economy strategies concerning valorization were provided by applying nanomaterials like agro-industrial wastes and by-products of food processing (94).

Various challenges in the food industry are met by nanotechnology. The use of nanotechnology meets the demand of the public regarding healthier and safer products. It provides the solution to several challenges, due to which it is regarded as a rapidly developing toolbox. The inclusion of nanomaterials in food products and packaging is because of their diversity and vast and tunable functionality. Bioactive fortification and prebiotics encapsulation helps to enhance the nutritional quality, and their antimicrobial and sensing properties help increase safety and confer novel sensorial properties. In this communication of food nanotechnology, matrix materials, particularly food-grade components, present and future production mechanisms, and in the fields of food preservation, safety and quality, bio-accessibility of nutrients and digestibility, current and potential applications have been mentioned in detail (95, 96). Nanostructures help promote safety of food as well as fortifying, along with bioavailability, processing, and encapsulation of active chemicals (97).

## Biocompatible Nanomaterials in Food Packaging

The use of nanomaterials in many foods and food sector-related disciplines offers significant potential properties, including processing, preservation, analysis, safety and delivery of food, and active and intelligent packaging. Innovative strategy requires the development of packaging having antimicrobial properties using polymer embedded nanomaterials. The commercial demand for silver nanoparticles is increasing due to their multiple beneficial uses in the market. The total use of these materials is in food-based products because of their antimicrobial properties. To counter a broad range of pathogenic organisms' silver nanoparticles are of prime importance as they are essential in evolving new antimicrobial agents (98). The use of developing technologies like as nanotechnology and antimicrobial packaging to effectively include biologically active chemicals and enhance planned functionalities i.e., smart biosensors is now trending in research and development for food packages manufacturing industries (99).

Recently, biological synthesis by using plant extracts to synthesize metal nanoparticles has been successfully performed. This investigation indicated the potential utilization of extracts from *P. serratum* leaves as an excellent bio-resource for the biosynthesis and diverse implementation of silver nanoparticles (AgNPs), more importantly in the packaging of food as an antibacterial agent and preservation against various foodborne pathogenic microbes. AgPVA nanofibers are used in antimicrobial packaging for food preservation in better manner (35).

Potential use of nanomaterials in various food science sectors has been found recently, including nano-sensor, packaging materials, encapsulated food components, etc. Nanomaterials based on polymers, liposomes, etc., are utilized in this sector due to their solubility, bioavailability, controlled release, etc. Antibacterial activity of the nanomaterials is associated with antibacterial mechanisms, including oxygen species, membrane damage, etc., in the domain of food science. The use of nanomaterials in food packaging is suitable as they exhibit enhanced barrier, mechanical and heat resistivity, and easy biodegradability (100).

Nanomaterials play an important part role in protecting, preserve foods and increase the shelf life. The use of pesticides, chemical or biological contaminants, and lifestyle changes directly affects food quality. Products having antimicrobial activity along with enhanced shelf life are produced by using microfibrillated cellulose nanomaterials (MCNs). Special attention is given to MCNs by researchers due to the prevention of microbial growth. It is possible to combine biopolymers/polymers with various MCNs, which act as a vehicle for individual particles and as a hybrid system allowing natural compounds and metallic nano-compounds. However, risk evaluation is needed to use nanoparticles in food packaging (101, 102).

Designing and developing various efficient food preservatives is one of the essential aspects of this field. However, due to the shortcomings of the bulk forms of such preservatives, research is in progress to find suitable alternatives to replace conventional modalities. In almost every aspect of food preservation, the approach has been made feasible with the intervention of nanotechnology. Over the past few decades, this domain of nanobiotechnology has been explored very well, and tremendous literature has been reported (103, 104).

Researchers have developed efficient nano-particles (NPs) having diverse applications. However, the literature available on food preservation based on nanotechnology does not include molecular perspectives involved in food preservation. To design edible coatings, nanotechnology and interface domain which is concerned with the physics of intermolecular and interfacial forces, contribute a lot, and there is a significant knowledge gap in this domain. To develop efficient NPs, identification of contributing factors at the nano and molecular levels is needed urgently. Moreover, it is essential to understand the impacts of NPs on health (105).

Various materials like nanoclay, nanosphere, nanowire, nanoceramic, nanoemulsion, nanotube, membrane, nanocapsule, and liposomal nanovesicle are used as packaging material for food. In packaged foods, biopolymers/natural hydrocolloids have been used because of their non-toxic nature and biodegradability to overcome environmental hazards. In addition to these positive effects, they also have some demerits like poorbarrier to moisture and harnessed mechanical properties. Due to their exceptional properties, interest in nanomaterials has been increased over the past few years (106). As they are capable of increasing thermal, mechanical, and gas barrier properties, they are used as precedent in food packaging without compromising their non-toxic and biodegradable ability. In food packaging, nanoparticles of zinc oxide, titanium dioxide and silver, kaolinite, montmorillonite (MMT), and coated silicate are used. They are considered important because films coated with these nanomaterials provide an excellent barrier against carbon dioxide, oxygen, and flavor compounds. They also possess antimicrobial activity, oxygen scavenging capability, and temperature tolerance. The most challenging task regarding the production of such nano-composites is their complete distribution within the polymer matrix and their compatibility. Therefore, enhanced performance of nano-packaging materials is demanded increasingly, including degradability, mechanical stability, and effectiveness of antibacterial property (107).

In the food sector importance of nanotechnology is increasing, particularly in food packaging and safety. Improvement in the barrier properties of packaging materials is expected with the incorporation of nanomaterials into packaging. The use of valuable raw materials and the generation of waste will also get reduced. In fresh fruits and food is protected from off-flavors and odors, lipids, gases, and moisture by the use of edible nanolaminates such as starch for encapsulation of pre and probiotics, vitamins, and drug delivery purposes natural biopolymers such as polysaccharides of nano-size can be used (108–110). As the process is time-consuming and laborious, these are the most critical problems in the food sector regarding the analysis of food quality control. To facilitate the preparation and analysis of food samples state of the art devices and methods are being developed. To detect microorganisms and contaminants, developing nano-sensors is an excellent application of food nanotechnology (111).

Significant barrier properties and catalytic, mechanical, optical, and antimicrobial properties can be induced into packaging with the help of nanotechnology. The majority of the packaging incorporating nanotechnology available in the market is based on silver nanoparticles (AgNPs) and nanoclay. Others, like nano-zinc oxide (ZnO) and titanium, share less in the current market. The use of nanomaterials in current food packaging is to induce antimicrobial function and enhance barrier functions. Thus, shelf life and freshness of the food gets enhanced (112).

## Bioactive Retention and Bioavailability of Nanocapsules and Gastric Conditions After Processing

The bioactive component's retention and bioavailability of nanocapsules made from different nanomaterials along with gastric conditions after processing in gut can be viewed in Table 2.

**Table 2:** Comprehensive insight to the bioactive component's retention and bioavailability of nanocapsules.

**Nanomaterial**	**Nanomaterial structure**	**Average size (nm)**	**Synthesis of food nanomaterial**	**Oral administration**	**Interaction with human organ/cells**	**Biocompatibility assessment**	**References**
Nanocapsule	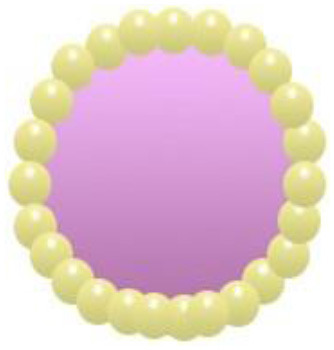	< 0.2 μm	Sodium alginate concentration is used to prepare nanoencapsulated quercetin	Nanoencapsulated bioactive compound quercetin	Prevent low gastric pH and during the digestion process optimize their release	Showed absent or low toxicity *in vitro* in different cell models and improved the bioavailability	(113)
Nanoliposomes	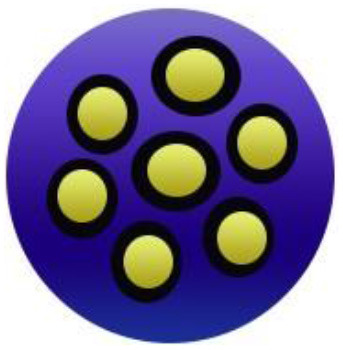	10 nm	Prepared by scattering the lipids in media that is aqueous and by purifying and analyzing	Nanoencapsulated curcumin	Interact with intestine by bile salts without the dispersion owing to their extra small size	Enhanced nutrients' bioavailability, safety of food, and improved bioaccessibility	(114)
Starch nanoparticles	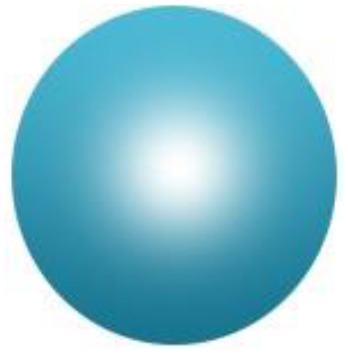	322.7 nm	Obtained from lotus stem	Nanoencapsulated catechin	Provide controlled intestinal release	Availability of high bioactive compounds, prevent various diseases	(75)
Nanostructured lipid capsules	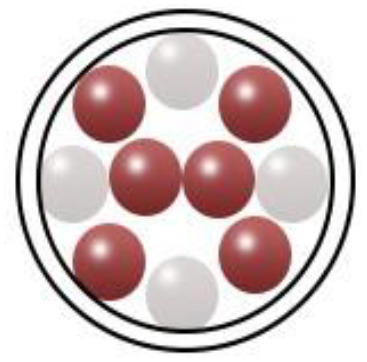	50–300 nm	Formed by interaction with various polyphenols such as catechin	Nanoencapsulation of polyphenols	Enhanced oral bioavailability and interact with gastrointestinal tract to gain high stability	Provide physiological treatment of several organs by acting as dietary supplements	(115)

## Current Developments

Recent developments are mainly based on medicine, among which stand out Amphotericin B which is a bioactive against fungal infections, and is being developed using Liposomes-nanotechnology approach by Gilead Sciences International Ltd., Uxbridge, UK, in form of suspension and marketed as AmBisome. Inactivated hepatitis A virus is being developed using virosomes-nanotechnology approach, and sold as suspension form vaccine under brandname Epaxal by Crucell Spain SA Madrid. A very common nano-medicine for iron deficiency anemia, Venofer is basically a nano-complex of Iron sucrose (iron (III)-hydroxide sucrose, is developed by Vifor in France and distributed to whole world (116).

## Legislations and Regulations for Nano-Products

Environmental legislation and rules address the usage, reusing, recycling, and certification of package materials. All food, feed, food packaging developing and waste managing have to follow 13th version of US EPA's Waste Reduction Model (117). In USA, Food and Drug Administration regulates and legislates the nanotechnology incorporated food, feed itself and packaging be it edible or non-edible also degradable or non-degradable. In 2017 this acclaimed organization developed rules and binding guidelines for use of nanotechnology and nanomaterials in products, packaging or systems and defined to investigate items as unique in existence and usage targeting case to case variations. Every food and drug developed by using nanotechnology must pass by FDA, otherwise could not be brought to market as to avoid health problems (118). European Union Observatory for Nanomaterials legislates alongwith other regulatory bodies on which products are safe as nanoproducts after prel-launch clinical investigations in UK and EU countries through Registration, Evaluation, Authorisation and Restriction of Chemicals (REACH) and Classification, Labeling and Packaging regulation (119).

## Health and Safety Concerns, Toxicity, and Public Perception for Using Nanotechnology in Foods

The migration of nanomaterials in the foodstuff is increasing as it can pose serious risks. Their market growth is hindered by insufficient information about the assessment of environmental and human safety. To overcome this difficulty, it is believed by the public that legislation from the government is essential. The characteristics and functions of significant nanomaterials commonly applied in food packaging, including their present and potential products, migration research, safety issues, and public concerns, are significant drives for nanomaterials development (81, 120). Nano-foods is a burgeoning field, yet toxicity of nano-materials is a major concern, a study by De Angelis and team maintained that nanoporous silicon based nano-particles of 2 nm pore size posed no histopathological injury in model mouse and exhibited zero toxicity; similar results were depicted by Park and co-scientists regarding toxicity of luminescent silver nanoparticles (121, 122).

Spoilage is a significant concern regarding food due to improper packaging technologies. Food packaging is expected to improve by nanotechnology. Packaging materials based on nanotechnology have unique properties, including antimicrobial potential, oxygen scavengers, barriers to the gas, or moisture, etc. Shelf life gets increased by using nanomaterials in food packaging without any unwanted change in its quality. The use of nano-based packaging of food is still in its initial stages, and hence, this review focuses on advances and an overview of the current status in the field. Various attempts have also been made to address safety and toxicity-related issues regarding public perceptions about nanomaterials and essential research areas in the field. The knowledge of positive and negative aspects of this technology will therefore define their acceptability as a sustainable material for packaging food (19, 123). Perception about nanotechnology in research published by Isabella and her team, depicts a negative notion in the minds of females to use it in cosmetics and dietary components as compared to males, so basically its application area of nanotechnology utility that concerns, simultaneously females were more keen to learn about the impact of nanotechnology as compared to males and they wanted to see it in product information label as if nanotechnology was involved at any level during processing (124). The expansion of awareness about scientific implications and reduction of negative perception about nanotechnology applications in daily life usage can be done by knowledge dissemination through events and community participation inclination in such activities to highlight benefits and risks of nanoparticles. Consumers are concerned about staying updated on the risks and benefits of using nanofoods (125).

## Conclusion

Nanotechnology applications are proliferating in food science and industry disciplines, which are among the fastest-growing and most promising areas of nanomaterial/nanotechnology applications. In the food sector, the development of innovative applications depends on advanced strategies like nanotechnology. Soon, biocompatible nanomaterials are expected to increase and therefore it has become an imperative technology for human exposure, especially by food consumption. Bio nano-composite materials open a door for the usage of the novel, high-performance, lightweight, and ecofriendly materials instead of traditional, non-biodegradable plastic packaging materials. Environmental hazards can be resolved due to the non-toxicity and biodegradability of various biopolymers such as polysaccharides like carboxymethyl cellulose (CMC), chitosan (CS), cellophane, and starch. Nanotechnology is an innovative technology and holds the potential to enhance food quality and safety. Biocompatible nanomaterials are being utilized in numerous areas of the food industry to explore their non-toxicity effects on the human body. There is a dire need for global standard legislations and testing protocols for nanoproducts.

## Future Recommendation

Through novel applications of nanotechnology, nanomaterials possess the excellent potential to increase the supply of food, including the absorption of nutrient and bioactive as well as systems for delivery; functionality of ingredients; enhanced colors and flavors; detection and control of microbes, allergens, and contaminants; and properties and performance of food packaging. Further studies are still needed to overcome important safety challenges and provide information on the risks and hazards of nanomaterials used in the food industry. The fundamental aspects of preservation of food future direction, molecular aspects, safety, and applicability of nano-preservatives should also be discussed.

## Author Contributions

MR, BS, and AR: methodology. SI, AS, and SS: validation. AM, AA, and UR: investigation. UR, SI, and SS: resources. MR, BS, SZ, AM, AA, UR, and AR: data curation. SS, MR, UR, BS, AM, AA, and AR: writing—original draft preparation. MR, BS, AM, AA, AR, UR, and SS: writing—review and editing. SI, UR, and AS: visualization. SI and AS: supervision and project administration. SI: funding acquisition. All authors have read and agreed to the published version of the manuscript.

## Funding

This work was funded in part by the USDA/NIFA through the Agricultural Research Program at North Carolina Agricultural and Technical State University (Evans-Allen Program, project number NC.X-291-5-15-170-1) and by an 1890 Capacity Building Program Grant (No. 2020-38821-31113/project accession No. 021765). SI would like to acknowledge the support of the Agricultural Research Station at North Carolina Agricultural and Technical State University (Greensboro, NC 27411, USA). This research was funded, in part, by grants (project Nos. NC.X337-5-21-170-1 and NC.X341-5-21-170-1) from the National Institute of Food and Agriculture (NIFA). Its contents are solely the responsibility of the authors and do not necessarily represent the official views of NIFA.

## Author Disclaimer

The contents are solely the responsibility of the authors and do not necessarily represent the official views of NIFA.

## Conflict of Interest

The authors declare that the research was conducted in the absence of any commercial or financial relationships that could be construed as a potential conflict of interest.

## Publisher's Note

All claims expressed in this article are solely those of the authors and do not necessarily represent those of their affiliated organizations, or those of the publisher, the editors and the reviewers. Any product that may be evaluated in this article, or claim that may be made by its manufacturer, is not guaranteed or endorsed by the publisher.
